# Psychometric Evaluation of the Polish Language Version of the Sleep Disturbance Scale for Children (SDSC)—A Pilot Study

**DOI:** 10.3390/jcm14072458

**Published:** 2025-04-03

**Authors:** Małgorzata Jączak-Goździak, Oliviero Bruni, Marcin Żarowski

**Affiliations:** 1 Department of Developmental Neurology, Poznan University of Medical Sciences, 61-701 Poznan, Poland; 2 Department of Developmental and Social Psychology, Sapienza University of Rome, 00185 Rome, Italy; oliviero.bruni@uniroma1.it

**Keywords:** sleep disorders, SDSC scale, Polish validation of SDSC

## Abstract

Sleep disorders in children and adolescents are common, affecting approximately 25–50% of children worldwide, yet they remain insufficiently researched. These sleep abnormalities, especially during developmental stages, can lead to various consequences, including emotional and behavioral disorders, academic challenges, mood disorders, and metabolic issues such as obesity. **Background/Objectives**: The study aimed to psychometrically evaluate a tool for examining sleep disorders in Polish children. **Methods**: The study involved a randomly selected sample of 42 children, all aged 10, from two primary schools in Poland: one located in a city with over 100,000 inhabitants and the other in a smaller town. Parents were asked to complete the Sleep Disturbance Scale for Children (SDSC) along with a sociodemographic survey. The study assessed reliability using Cronbach’s alpha (α) and evaluated the correlation between individual domains using Spearman’s rank correlation coefficient (Rs). **Results**: The pilot group demonstrated very good internal consistency for the entire SDSC scale, with a Cronbach’s α value of 0.89, and suitable values for the individual subscales (ranging from 0.69 to 0.83). Additionally, there was a positive correlation between the individual subscales. **Conclusions**: While this pilot study requires validation with a larger patient group, the results suggest that the SDSC scale could be an effective tool for screening sleep disorders among Polish children.

## 1. Introduction

Sleep disorders in children are a common problem [1,2,3], but still insufficiently recognized and treated [3]. Sleep-related issues are estimated to affect 25–50% of children worldwide [4], and these abnormalities significantly affect the functioning of the entire family, especially caregivers [5]. The type of sleep disorders changes with age: in younger children, problems with sleeping through the night predominate; in school-age children, parasomnias occur in particular; and in teenagers, there is a risk of insomnia, circadian rhythm disturbances, and excessive daytime sleepiness [4].

**Figure 1:**
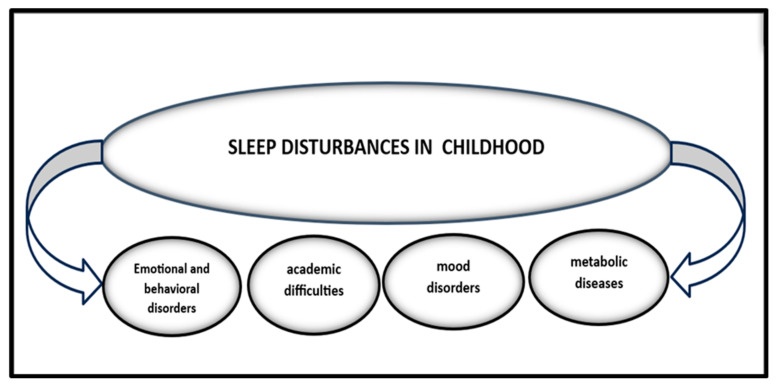
Consequences of sleep disturbances in childhood.

The childhood and teenage period is a period of maturation of the nervous system and the development of the circadian rhythm and proper sleep architecture [6]. Sleep disorders from this period affect functioning in later life [6] and have consequences in many domains: emotional, behavioral, cognitive, academic, and physical [4]. In children and adolescents, sleep problems lead to school difficulties (impaired memory, attention, and problem-solving), mood disorders (depression, anxiety disorders, and antisocial behavior), and metabolic disorders, including obesity [7,8,9,10] (Figure 1).

In the literature, there are several tools (mainly questionnaire-based) for assessing sleep quality and sleep-related problems [2]. They have been used in screening tests for years and provide repeatable, standard measurements using an inexpensive and accurate method [2]. Among the various screening survey tools, we can distinguish between the following:

1. Questionnaires based on parent reports;
2. Questionnaires for self-reporting by the patient [5].

The most popular questionnaires for parents include the following:

- Children’s Sleep Habits Questionnaire (CSHQ): This tool is designed for parents of children aged 4 to 10 and helps screen the most common sleep disorders in this age group. It addresses issues such as difficulties falling asleep, delayed sleep onset, insufficient sleep duration, sleep-related anxieties, nighttime awakenings, parasomnias, and sleep-related breathing disorders [5,11].
- Sleep Disturbance Scale for Children: A brief screening questionnaire to identify sleep disorders in children and adolescents aged 6 to 15 [1,5].

Available self-assessment questionnaires include the following:

- Children’s Sleep Comic (CSC): Intended for children aged 5 to 10, this questionnaire allows them to report on their sleep habits and age-specific sleep problems [5,12].
- Sleep Self-Report Scale (SSRS): A validated tool for assessing sleep disorders in children aged 7 to 12 [5,13].
- Epworth Sleepiness Scale for Children (ESS-C): This version of the Epworth Sleepiness Scale, adapted for children aged 6 to 19, evaluates daytime sleepiness [5,14].
- Nightmares Effects Questionnaire (NEQ): This questionnaire assesses the impact of nightmares on daytime functioning in adolescents. It consists of 30 items that evaluate six factors: emotion regulation, stress, aggressiveness, depression, attentiveness/concentration, anxiety, and hyperactivity [5].

To our knowledge, there has yet to be a validated sleep assessment scale for pediatric patients in Poland (neither self-report nor parent report), and due to the universality of the problem, the preparation of such a tool is necessary.

## 2. Materials and Methods

The Sleep Disturbance Scale for Children was selected for validation, as it is one of the few that meets all the methodological psychometric criteria [15] and has high internal consistency determined by Cronbach’s alpha index of 0.79 in the control group and 0.71 in the study group [1,16]. The Sleep Disturbance Scale for Children was constructed and validated in Italy in 1996 [1].

The SDSC scale is intended to be completed by parents of children aged 6 to 15 years [2]. The questions concern the period of the last six months [2,4,17]. The questionnaire takes approximately 10 min to complete [16]. The scale contains 26 questions; the answers are on the Linkert scale [2,4,5,16]. The first two questions about the total sleep time, where (1) 9–11 h, (2) 8–9 h, (3) 7–8 h, (4) 5–7 h, and (5) less than 5 h, and sleep latency—the time the child falls asleep after lying down: (1) <15 min, (2) 15 to 30 min, (3) 30–45 min, (4) 45–60 min, and (5) >60 min. The answers to the remaining 25 questions are marked on a 5-point scale (1—never; 2—rarely (once or twice a month or less often); 3—sometimes (once or twice a week); 4—often (3 to 5 times per week); and 5—always (every day)) [4].

The minimum value of points on the scale is 26, and the maximum is 130 [2]. The higher the total value, the more severe sleep-related problems [2,4]. The SDSC scale, apart from global verification of the degree of sleep disorders, allows for the assessment of the six most common groups of sleep disorders:

- Disorders of initiating and maintaining sleep (DIMS) assessment in questions 1, 2, 3, 4, 5, 10, and 11;
- Sleep breathing disorders (SBD) in questions: 13, 14, 15
- Disorders related to arousal (DA disorders of arousal) in questions 17, 20, 21
- Sleep–wake transition disorders (SWTD) in questions 6, 7, 8, 12, 18, 19
- Disorders of excessive sleepiness (DOES disorders of excessive somnolence) questions: 22, 23, 24, 25 and 26
- excessive sweating during sleep (SHY sleep hyperhidrosis) questions: 9 and 16 [4].

The subscales are consistent with the Association of Sleep Disorders Centers (ASDC) classification and appear more suited to diagnosing sleep disorders in children than the International Classification of Sleep Disorders (ICDS) categories [16].

The SDSC scale, as a useful screening tool, has been translated and validated into several languages: Italian, Portuguese, French, Flemish, Finnish, Persian, Chinese, Malaysian, Indonesian, Malayalam, and Spanish [4,17,18,19,20,21,22,23,24,25,26,27,28].

**Figure 2:**
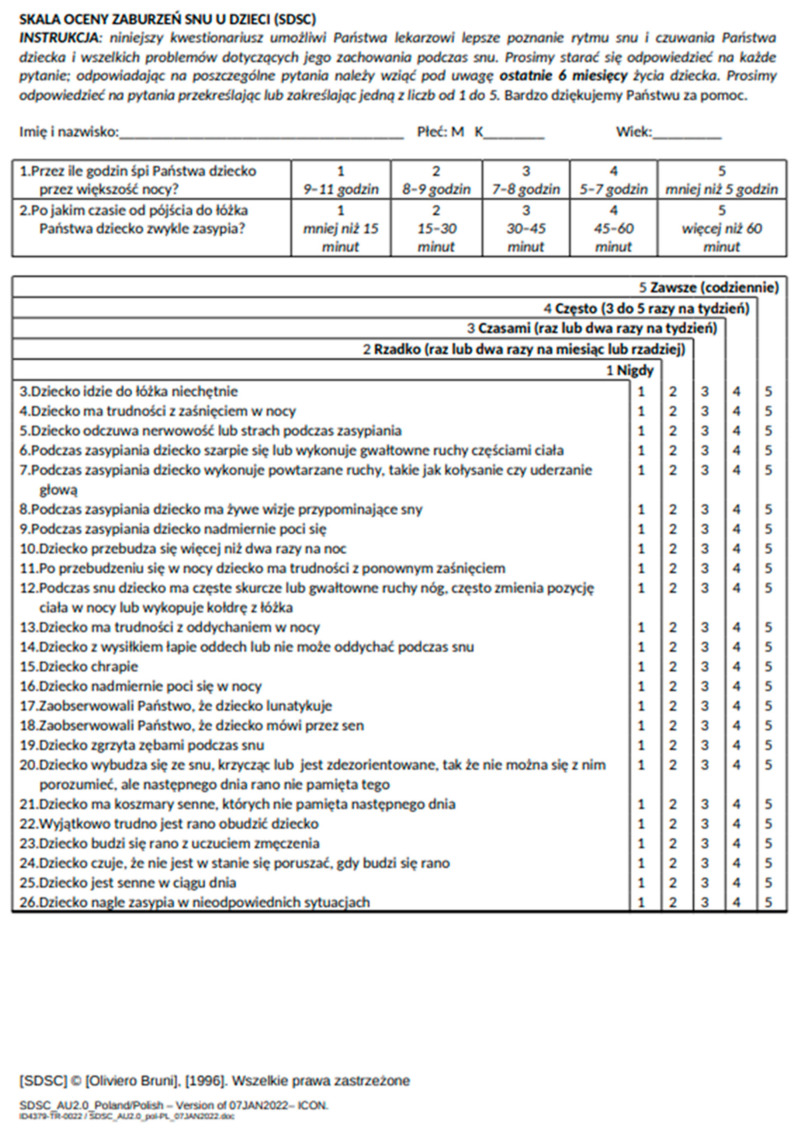
Sleep Disturbance Scale in Polish (SDSC).

In the validation study into Polish, the stage of translating the scale was omitted because after contacting Professor Oliviero Bruni (one of the authors) to obtain consent, we received from the author the certified Polish translation of the SDSC (Figure 2). Therefore, the translation step was unnecessary.

The pilot study of the validation of the SDSC scale in Polish was started after obtaining the consent of the Bioethics Committee of the Poznań University of Medical Sciences. The pilot was carried out among parents of ten-year-olds from two primary schools in the Poznań agglomeration—one in a city with over 100,000 inhabitants and one in a smaller town. After obtaining the consent of the school authorities, teachers in grades 3 and 4 provided parents with information forms, a sociodemographic survey, and SDSC scales in Polish to read and fill out anonymously. The age group of 10-year-olds was selected for the pilot study—as the middle group for the age purpose of the scale SDSC.

In the sociodemographic survey, parents were asked to provide the age and gender of the child, their education (primary, secondary, or higher), the number of inhabitants in the place of residence, and to indicate the diseases and medications taken by the child. We were also asked whether the child slept alone in the room and the bed, and information about sibling number and description: younger or older.

### 2.1. Characteristics of the Study Group

A total of 42 forms were qualified for the pilot study, where the SDSC scale was completed correctly by all the participants. The response rate for the forms was 60%. Two forms were excluded because the SDSC scale was not fully completed. The final analysis included responses from the parents of 22 girls and 20 boys, with the majority of the forms being filled out by mothers (34 forms) compared to fathers (8 forms). Twenty-seven children live in a city with over 100,000 inhabitants. Most children sleep alone in the room (28 vs. 14). 40 out of 42 children sleep alone in a bed. Thirty-one children have siblings, including 16 children who have older siblings. The minimum scale score obtained in the pilot group was 28, and the maximum was 74.

### 2.2. Statistical Analysis

Calculations were performed using Statistica 13.3 by TIBCO and PQStat v.1.8.6.122 by PQStat Software (2024). The level of significance was α = 0.05. The result was considered statistically significant when *p* < α. The consistency of the questionnaire was determined by examining the value of Cronbach’s alpha coefficient, both for the entire questionnaire and for individual domains. Correlations between individual domains were examined using Spearman’s Rs rank correlation coefficient.

## 3. Results

The analysis obtained an excellent internal consistency index for the entire scale—Cronbach’s α value of 0.89 and also good for the individual subscales: the lowest—0.69 for DIMS, the highest—0.83 for SHY (values for individual subscales are presented in Table 1:

In the pilot study, correlations between individual subscales and the entire scale were examined; the results are presented in Table 2:

A positive correlation was obtained between the individual subscales and the entire scale (0.36–0.78). Significant correlations (marked in bold in Table 2) were found between DIMS and DA, DIMS and DOES, SBD and SWTD, SBD and SHY, DA and SWTD and DA and SHY, SWTD and DOES, and SWTD and SHY. All the correlations are positive.

In the study, 16 (38%) children achieved a score > 39, a value that in the original research was the cut-off point for children requiring additional diagnosis of sleep disorders. This is quite a high percentage of people affected by sleeping problems and needs analysis in a larger group.

## 4. Discussion

A pilot study conducted with 10-year-olds demonstrated excellent psychometric properties of the SDSC scale in Polish, achieving a high Cronbach’s α value of 0.89 for the entire scale, as well as for all the subscales. This value is slightly higher than that reported in the original study (0.79) and comparable to other language studies (0.82–0.85) [2,4,16]. The SBD subscale yielded the highest Cronbach’s α value of 0.88, while the DIMS subscale had the lowest at 0.66. Notably, the Cronbach’s α value for the DA subscale was higher than in previous studies: 0.467 in Turkish, 0.39 in Spanish, 0.4 in Finnish, and 0.4 in Persian studies [2,4,23,24].

Consistent with the findings from the Spanish study, there was a positive and significant correlation between all the subscales and the overall scale, ranging from 0.36 to 0.78 [4]. Similarly to previous research [1,4,20,24], the highest correlations with the entire scale were observed for the DIMS, SWTD, and DOES subscales. In contrast to earlier studies in Spain and Turkey [4,24], this pilot study revealed significant correlations between several subscales (as indicated in bold in Table 2). Furthermore, we found a correlation of 0.44 between the DIMS and DOES subscales, which aligns with the Turkish study’s finding of 0.46 [24]. This suggests that individuals with difficulty falling asleep tend to experience more significant daytime sleepiness.

Certain limitations should be acknowledged in this study. First, the participant population consisted only of those who completed the entire survey. This may have introduced response bias, as parents of children with issues might have been more motivated to complete the questionnaire. Moreover, it is important to note that survey studies based on parental reports may underestimate specific sleep-related symptoms in children whose parents are unaware of these problems. Additionally, the study focused solely on healthy children, and future research should include a clinical group for comparison. The results from this clinical group should be evaluated against those obtained through objective research methods, such as actigraphy or polysomnography. Finally, the study needs to be validated with a significantly larger sample encompassing all age groups, which is part of the researchers’ plans.

## 5. Conclusions

The study is a pilot study and requires confirmation on a larger group of patients, but the results are promising. A larger study group is also necessary to establish an appropriate cut-off point for the population of Polish children. The SDSC scale is a perfect screening tool for diagnosing sleep disorders among Polish children. It allows the selection of children who will require additional tests.

## Figures and Tables

**Table 1:** Cronbach’s α value for the entire scale and individual subscales.

	DIMS	SBD	DA	SWTD	SHY	DOES	Total
Group size	42	42	42	42	42	42	42
Number of items	7	3	3	6	5	2	26
Mean	12.19	4.024	4.048	8.69	7.67	2.93	39.555
Standard deviation (SD) of the scale	3.44	2.09	1.68	3.06	2.97	1.553	10.57
Cronbach’s α value	0.69	0.85	0.80	0.75	0.80	0.83	0.89
−95% CI Cronbach’s	0.52	0.75	0.67	0.62	0.68	0.68	0.84
+95% CI Cronbach’s	0.81	0.91	0.89	0.85	0.88	0.91	0.93
Standard error (SE) of measurement	1.93	0.81	0.759	1.52	1.33	0.65	3.51
Average correlation	0.22	0.71	0.57	0.36	0.44	0.77	0.25
Cronbach’s alpha standardized (STD)	0.66	0.88	0.80	0.77	0.79	0.87	0.89

Explanations: DA: disorders of arousal; DIMS: disorders of initiating and maintaining sleep; DOES: disorders of excessive somnolence; SBD: sleep breathing disorders; SHY: sleep hyperhidrosis; SWTD: sleep–wake transition disorders.

**Table 2:** Matrix plot of correlations (Spearman Rs).

	DIMS	SBD	DA	SWTD	DOES	SHY	Total
DIMS	1.00						
SBD	0.13	1.00					
DA	**0.51**	0.17	1.00				
SWTD	0.30	**0.36**	**0.42**	1.00			
DOES	**0.44**	0.07	0.16	**0.47**	1.00		
SHY	0.28	**0.52**	**0.41**	**0.56**	0.20	1.00	
Total	**0.78**	**0.36**	**0.60**	**0.72**	**0.67**	**0.69**	1.00

Explanations: DA: disorders of arousal; DIMS: disorders of initiating and maintaining sleep; DOES: disorders of excessive somnolence; SBD: sleep breathing disorders; SHY: sleep hyperhidrosis; SWTD: sleep–wake transition disorders. Significant correlations marked in bold.

## Data Availability

The original contributions presented in this study are included in the article. Further inquiries can be directed to the corresponding author.

## Abbreviations

The following abbreviations are used in this manuscript:

SDSC	Sleep Disturbance Scale for Children
DIMS	disorders of initiating and maintaining sleep
SBD	sleep breathing disorders
SWTD	sleep–wake transition disorders
DOES	disorders of excessive somnolence
SHY	sleep hyperhidrosis
